# Total synthesis of rupestine G and its epimers

**DOI:** 10.1098/rsos.172037

**Published:** 2018-03-28

**Authors:** Abdullah Yusuf, Jiangyu Zhao, Bianlin Wang, Paruke Aibibula, Haji Akber Aisa, Guozheng Huang

**Affiliations:** 1 Key Laboratory of Plant Resources and Chemistry in Arid Regions, and State Key Laboratory Basis of Xinjiang Indigenous Medicinal Plants Resource Utilization, Xinjiang Technical Institute of Physics and Chemistry, Chinese Academy of Sciences, South Beijing Rd 40-1, Urumqi, 830011, People's Republic of China; 2 University of Chinese Academy of Sciences, Yuquan Rd 19 A, Beijing, 100049, People's Republic of China; 3 College of Chemistry and Environmental Science, Kashgar University, Xueyuan Rd 29, Kashgar, 844000, People's Republic of China

**Keywords:** rupestine G, RCM reaction, guaipyridine sesquiterpene

## Abstract

Rupestine **G** is a guaipyridine sesquiterpene alkaloid isolated from *Artemisia rupestris* L. The total synthesis of rupestine **G** and its epimers was accomplished employing a Suzuki reaction to build a terminal diene moiety. The diene was further elaborated into the desired guaipyridine structure by a ring-closing metathesis reaction. Over all, rupestine **G** and its three epimers were obtained as a mixture in a sequence of nine linear steps with 18.9% yield. Rupestine **G** and its optically pure isomers were isolated by chiral preparative HPLC and fully characterized by ^1^H ,^13^C NMR, HRMS, optical rotation value, and experimental and calculated electronic circular dichroism spectroscopy.

## 1. Introduction

**Figure 1.**
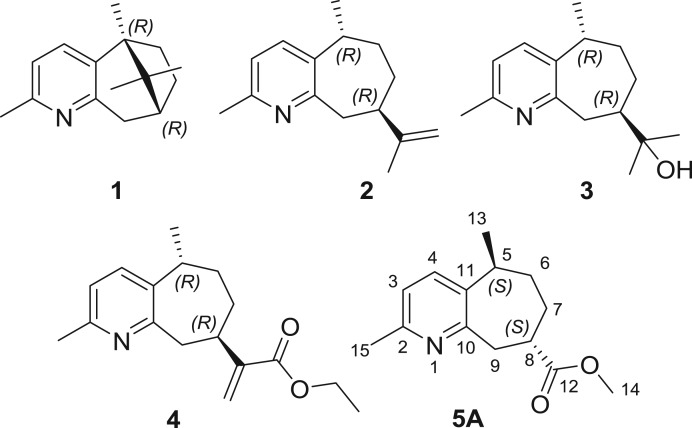
Structures of representative guaipyridine sesquiterpene alkaloids.

Guaipyridine sesquiterpene alkaloids are a family of natural compounds that share a unique structure consisting of a fused pyridine ring and seven-membered carbocycle [1]. For example, patchoulipyridine (**1**, figure 1) and epiguaipyridine (**2**, figure 1) were first isolated from the essential oil of *Pogostemon patchouli* Pellet by Büchi *et al*. in 1966 [2]. Another representative guaipyridine alkaloid, cananodine (**3**, figure 1), was isolated from the fruits of *Cananga odorata*. Cananodine shows potent activity against Hep G2 cell lines with a sub-micromolar IC_50_ value [3]. Recently, a series of guaipyridine sesquiterpene alkaloids, namely rupestine A–M (**4:** rupestine **A; 5A:** rupestine **G,** figure 1) were discovered by our group from *Artemisia rupestris* L., a well-known traditional Chinese medicinal plant used for detoxification, antitumour, antibacterial and antiviral activity, and for protecting the liver [4–7]. Owing to their structural similarities when compared with cananodine, it is suggested that rupestines might also possess promising cytotoxic activity. Unfortunately, biological evaluations of these alkaloids were limited by their scarce availability from natural sources. Hence, their scarcities and their unique structural features render them worthy targets for their total synthesis.

**Scheme 1.**
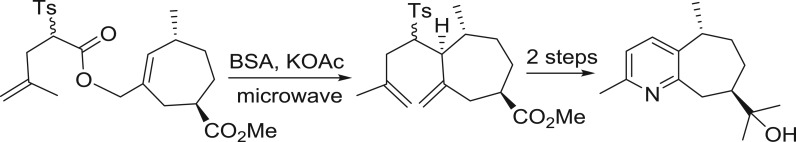
Craig's strategy for synthesis of (+)-cananodine.

The first synthesis of guaipyridine sesquiterpene was accomplished by Büchi *et al.* [2]. Exposure of β-patchoulene to hydrazoic acid in the presence of H_2_SO_4_, followed by dehydrogenation in hot 1-methylnaphthalene over Pd/C produced patchoulipyridine (**1**) as the major product. Van der Gen *et al.* [8] isomerized the 1,5-double bond of guaiol to obtain the desired isomer with a 4,5-double bond, which was further oxidized with ozone and treated with hydroxylamine. By this way, the 5-epimer of epiguaipyridine (**2**) was synthesized. It should be noted that the absolute configuration of van Der Gen's synthetic product is different from that of the ‘natural' one proposed by Büchi *et al.* [2]. Since neither β-patchoulene nor guaiol are commercially available, it is inevitably necessary to isolate them before the initiation of the synthesis. Decades later, Craig & Henry [9] applied a microwave-assisted decarboxylative Claisen rearrangement to synthesize (+)-cananodine in 2006 (scheme 1).

**Scheme 2.**
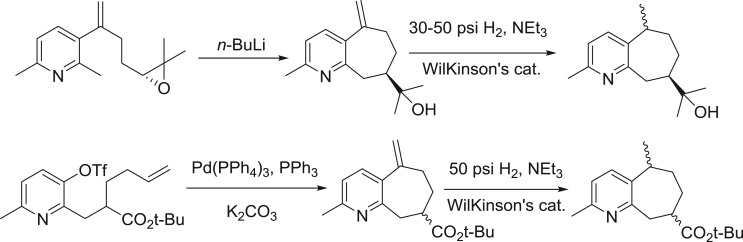
Vyvyan's strategy for synthesis of rupestines.

Another strategy was to build the seven-membered ring of guaipyridine compounds using derivatives of pyridine as the starting material. Applying this strategy, the Vyvyan group explored a base-promoted epoxide-opening and an intramolecular Heck cyclization to build the guaipyridine core (scheme 2) [10–12]. This approach subtly uses cheap and commercially available chemicals to launch the synthesis and deserves to be further developed.

## 2. Results and discussion

Natural rupestines are usually isolated as isomeric compounds, with different configurations at 5- and 8-positions. For example, rupestine **B** and **C**, rupestine **H** and **I** as well as rupestine **L** and **M** are natural isomeric compounds (electronic supplementary material, figure S0) [7]. Rupestine **E** was once erroneously assigned as its (*5R*,*8R*)-isomer, i.e. rupestine, a compound that has actually not been isolated from the natural plant [4–7]. In view of the confusion regarding the structural elucidation of isomers of rupestine, preparation of all isomers would be beneficial for the confirmation of their individual structural characterizations and biological evaluations. Thus, we chose a nonstereoselective route to provide the four isomers in a single reaction.

**Scheme 3.**
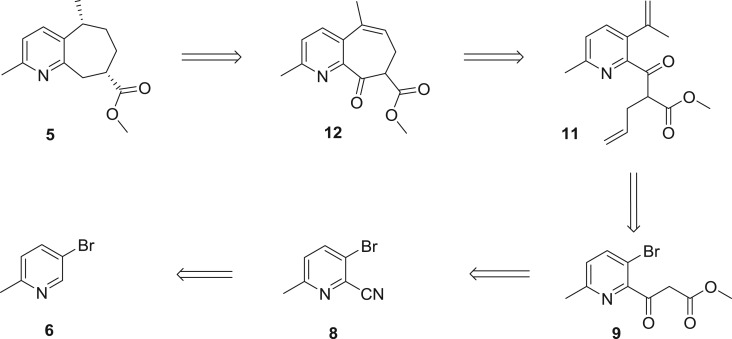
Retrosynthetic analysis of (±)-rupestine **G**.

Retrosynthetically, rupestine **G** (**5A**) could be obtained by reduction of intermediate **12**. Compound **12** was envisaged to be constructed by a ring-closing metathesis (RCM) reaction from the substituted diene **11**. By application of a Suzuki cross-coupling reaction and alkylation, compound **11** could be obtained smoothly starting from compound **9**. Furthermore, compound **9** could be accessible by decarboxylative Blaise reaction of picolinonitrile **8**, which could be rapidly prepared from commercially available 5-bromo-2-picoline **(6)** (scheme 3).

**Scheme 4.**
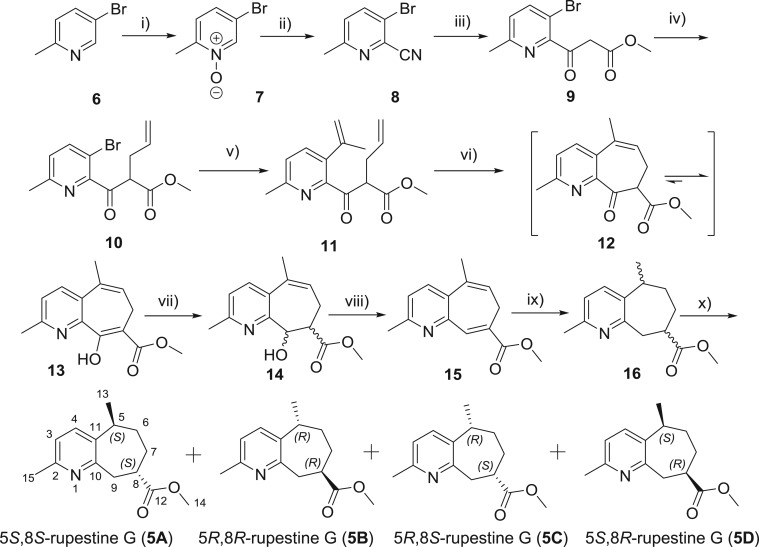
Synthesis of rupestine G and its epimers. Reagents and conditions: (i) *m*-CPBA, CH_2_Cl_2_, r.t., overnight, 92.7%; (ii) trimethylsilyl cyanide, triethylamine, MeCN, reflux, 12 h, 85.1%; (iii) a. ZnCl_2_, potassium methyl malonate, *N*,*N*-diisopropylethylamine, 1,2-dichloroethane, reflux, 16 h; b. 6N HCl, reflux,1 h, 82.1%; (iv) 3-bromopropene, EtONa, EtOH, r.t., overnight, 97.3%; (v) isopropenylboronic acid pinacol ester, Pd(Ph_3_P)_4_, 1,4-dioxane/H_2_O (*v/v *= 3:1), reflux, 3 h, 91.8%; (vi) Grubbs II, CH_2_Cl_2_, reflux, 12 h, 53.3%; (vii) NaBH_4_, MeOH, r.t., 1 h, 77.5%; (viii) MsCl, pyridine, 60°C, 3 h, 86.6%; ix) Pd/C, H_2_,MeOH, r.t., 5 h, 91.4%; (x) isolation by preparative HPLC, hexane/EtOH (*v/v *= 98:2).

The final synthesis strategy of rupestine **G** is shown in scheme 4. The 2-cyanopyridine **8** was readily prepared by *m*-CPBA oxidation and modified Reissert–Henze reaction from 5-bromo-2-methylpyridine (**6**) following the method developed by Fife [13–15]. The methyl nicotinoylacetate **9** was obtained from decarboxylative Blaise reaction of **8** with potassium methyl malonate in 82% yield [16–19]. Treatment of **9** with allyl bromide in the presence of sodium ethoxide provided **10** in 97% yield. After screening several Suzuki cross-coupling conditions, it was found that using isopropenylboronic acid pinacol ester, instead of unstable prop-1-en-2-ylboronic acid, gave compound **11** in 92% yield [20–24]. The pivotal RCM reaction catalysed by the Grubbs II catalyst was carried out to build the seven-membered ring in 53% yield [25–28]. According to the NMR data, the ring-closed product favoured the enol form **13** rather than the keto form **12**, although both of the two tautomers were detectable on thin layer chromatography. The moderate but still acceptable yield of RCM reaction probably was the result of an undesired intermolecular reaction. The reaction in low concentration provided less intermolecular by-product, but also low conversion of the starting material **11**. To sum up, the six-step reaction successfully constructed the frame of guaipyridine.

Compound **13** was then reduced by NaBH_4_ in MeOH. Theoretically, reduction of compound **13** would present one additional chiral carbon in the product, hence we did not purify the compound **14** but directly dehydrated it with MsCl in pyridine at 60°C and obtained diene **15** in a total yield of 67.1% in two steps. Hydrogenation of compound **15** catalysed by Pd/C in MeOH gave rupestine **G** and its epimers as a mixture in an overall 91.4% yield. Thus, from 5-bromo-2-picoline (**6**), the desired target was obtained in an overall 18.9% yield.

The mixture (46.4 mg) was first isolated on a preparative TLC to give two pairs of diastereoisomers (31.0 and 10.6 mg, i.e. **16a** and **16b**, respectively). These two pairs of compounds were further separated by chiral separation with a Shimadzu LC-20A preparative HPLC, to give four optically pure isomers.

**Figure 2.**
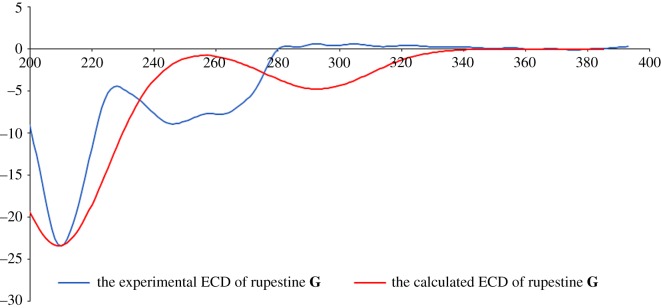
The experimental ECD and calculated ECD of rupestine **G**.

The structures of these four isomers were intensively elucidated by extensive analysis with ^1^H NMR, ^13^C NMR, high-resolution-electrospray ionization--mass spectrometry (HR-ESI-MS) and electronic circular dichroism spectroscopy (ECD) (figure 2).

The HR-ESI-MS of compound **5A** at *m/z* 234.1490 (M + H)^+^ tallies with the reported natural product rupestine **G** (*m*/*z* = 234.1509) [4]. The ^1^H and ^13^C NMR data are identical to the previously published data [4]. However, the optical rotation value is [*α*]_D_^20^ = −41.0 (*c* = 0.10, MeOH), which differs from that obtained previously {(*α*)_D_^20^ = –16.0 (*c* = 0.03, MeOH)}. It is presumed that the previous measurement of the optical rotation value at low concentration resulted in some inaccurate data, as usually the greater order of magnitude of concentration renders it less susceptible to experimental error [9]. The CD spectra of **5A** show a similar CD pattern with the calculated data, i.e. a negative Cotton effect (CE) near 215 nm and a negative CE in the 230–280 nm region, which verifies that the absolute configuration of compound **5A** is 5*S,* 8*S*. The ^1^H and ^13^C NMR data of compound **5B** are identical to **5A**, but the CD spectra are opposite to those of **5A**. Thus the compound **5B** is confirmed as 5*R,*8*R*-rupestine **G.** With the same virtue, compound **5C** and **5D** are confirmed as 5*R,*8*S*-rupestine **G** and 5*S,*8*R*-rupestine **G**, respectively.

## 3. Conclusion

In summary, we have achieved the first total synthesis of rupestine **G** and its epimers in a sequence of nine linear steps starting from commercially available 5-bromo-2-picoline. Notable transformations include a decarboxylative Blaise reaction between potassium methyl malonate and picolinonitrile and a Suzuki reaction to induce an isopropenyl group. The construction of the seven-membered ring was accomplished by a RCM reaction. Hydrogenation of the diene moiety finalized the synthesis of rupestine **G** and its epimers. Preparative HPLC obtained four optically pure isomers and their structures were fully characterized by ^1^H, ^13^C NMR, HRMS, optical rotation value, and experimental and calculated ECD. The synthetic approach demonstrated herein would be equally effective for the synthetic preparation of other guaipyridine sesquiterpene alkaloids. Biological evaluations of rupestine **G** and its epimers are ongoing and will be published in due course.

## 4. Material and methods

All reactions were performed in oven-dried flasks. Reagents and solvents were purchased from commercial vendors and used as received. Reaction progress and purity of the compounds were monitored by TLC. ^1^H and ^13^C NMR spectra were recorded on a Varian VNMRS 600 spectrometer and Varian 400-MR in CDCl_3_ or DMSO-*d_6_* with TMS as an internal reference. The HR-ESI-MS data were collected with a QStar Elite mass spectrometer. Melting points were measured with a BUCHI B-540 melting point apparatus. Semi-preparative HPLC was conducted on a Shimadzu LC-20A instrument, with UV detection, using a CHIRALPAK ID-Lot (No. ID00CE-QI011) column. As mobile phase, 98% *n*-hexane in ethanol was used (HPLC grade, Merck, Germany). The optical rotations were recorded on a Rudolph RS Autopol VI automatic polarimeter. ECD spectra were measured in EtOH on a JASCO J-810 spectropolarimeter (Jasco, Tokyo, Japan). ECD calculations were performed by TmoleX 3.4 software (COSMOlogic GmbH & Co. KG, Germany) [29–32]. Absolute configuration was assigned by using optical rotation spectra, circular dichroism spectroscopy and time-dependent density functional theory calculations at BP/TZVPP level. The ground-state geometries were optimized with density functional theory calculations. All atoms were estimated with the basis set def-TZVP and the functional BP. Electronic circular dichroism corresponding to the optimized structures was calculated using the TDDFT method at BP/def-TZVP level. The results were subsequently optimized by the Gaussian method.

### 4.1. Synthesis and characterization data of products

#### 4.1.1. 5-Bromo-2-methylpyridine 1-oxide (**7**)

To a solution of 5-bromo-2-methylpyridine (2.00 g, 11.7 mmol) in chloroform (30.0 ml) was added *meta*-chloroperoxybenzoic acid (85.0%, 2.85 g, 14.0 mmol, 1.20 eq.) in portions. After the addition, the reaction mixture was stirred at room temperature overnight and quenched with 10% sodium bisulphite solution, followed by the addition of 2 M aqueous sodium carbonate to neutralize the acid. After filtration, the aqueous layer was extracted with CH_2_Cl_2_ (20.0 ml ×3), and the combined organic portions were dried over magnesium sulfate, filtered, and concentrated *in vacuo* to give 5-bromo-2-methylpyridine 1-oxide (2.04 g, 92.7%) as a white solid; m.p. 121.9–122.1°C; (lit [33], 119.5–120.1°C); IR (neat) *v*_max_ 3043, 2961, 1600, 1486, 1444, 1087, 828, 800 cm^−1^.

#### 4.1.2. 3-Bromo-6-methylpicolinonitrile (**8**)

To a solution of 5-bromo-2-methylpyridine 1-oxide (1.00 g, 5.38 mmol) in acetonitrile (27.0 ml) was added trimethylsilyl cyanide (2.13 g, 21.5 mmol, 4.00 eq.) and triethylamine (2.23 ml, 16.1 mmol, 3.00 eq.). The reaction mixture was stirred at 100°C for 12 h. After cooling to room temperature, the solvent was evaporated off *in vacuo*. The residue was purified by Combiflash (eluted with 0 ∼ 50% ethyl acetate in petroleum) to give 3-bromo-6-methylpicolinonitrile (0.89 g, 85.1%) as a white solid; HR-ESI-MS, Calcd 195.9636, found [M + H]^+^ = 196.9703. m.p. 94.9–95.2°C (lit [34], 93.8–94.6°C); IR (neat) *v*_max_ 3044, 2981, 2234, 1575, 1443, 1032, 844, 697 cm^−1^; ^1^H NMR (400 MHz, CDCl_3_) *δ* 7.98 (d, *J* = 8.2 Hz, 1H), 7.24 (d, *J* = 3.0 Hz, 1H), 2.57 (s, 3H).

#### 4.1.3. Methyl 3-(3-bromo-6-methylpyridin-2-yl)-3-oxopropanoate (**9**)

To a solution of 3-bromo-6-methylpicolinonitrile (5.50 g, 27.9 mmol) in 1,2-dichloroethane (100 ml) were added zinc chloride (60.0%, 7.59 g, 33.5 mmol, 1.20 eq.), and potassium methylmalonate (5.22 g, 33.5 mmol, 1.20 eq.), and *N,N*-diisopropylethylamine (1.38 ml, 8.37 mmol, 0.30 eq.) then the mixture was stirred at reflux for 16 h. Then 5.00 ml of 6 N hydrochloric acid was added to the mixture, which was stirred at reflux at 90°C for 1 h. The reaction mixture was cooled to 20°C, and neutralized with 2 M aqueous sodium carbonate. The organic layer was concentrated *in vacuo*. The resulting residue was purified by silica gel column chromatography (ethyl acetate/petroleum, 1/20) to obtain the title compound (6.18 g, 82.1%) as a white solid; m.p. 73.7–74.1°C; HR-ESI-MS, Calcd 270.9844, found [M + H]^+^ = 271.9908; IR (neat, film) *v*_max_ 2951, 2844, 1747, 1707, 1462, 1379, 1058, 1018, 828, 654 cm^−1^; ^1^H NMR (400 MHz, CDCl_3_) *δ* 7.87 (d, *J* = 8.2 Hz, 1H), 7.14 (d, *J* = 8.2 Hz, 1H), 4.13 (s, 2H), 3.71 (s, 3H), 2.52 (s, 3H); ^13^C NMR (100 MHz, CDCl_3_) *δ* 193.16, 168.26, 156.38, 148.83, 142.88, 127.11, 115.38, 52.02, 46.13, 23.52.

#### 4.1.4. Methyl 2-(3-bromo-6-methylpicolinoyl)pent-4-enoate (**10**)

To a solution of methyl 3-(3-bromo-6-methylpyridin-2-yl)-3-oxopropanoate (1.00 g, 3.21 mmol) in ethanol (30.0 ml) were added sodium ethoxide (0.26 g, 3.85 mmol, 1.20 eq.) and 3-bromopropene (0.33 ml, 3.85 mmol, 1.20 eq.), then the mixture was stirred at room temperature overnight. Afterwards, the reaction mixture was concentrated *in vacuo*. The resulted residue was purified by silica gel column chromatography (ethyl acetate/petroleum, 1/20) to obtain the title compound (0.97 g, 97.3%) as a yellow oil. HR-ESI-MS, Calcd 311.0157, found [M + H]^+^ = 312.0221; IR (neat, film) *v*_max_ 3078, 2950, 2848, 1744, 1710, 1573, 1435, 1246, 1194, 1166, 968, 828 cm^−1^; ^1^H NMR (400 MHz, CDCl_3_) *δ* 7.86 (d, *J* = 8.2 Hz, 1H), 7.13 (d, *J *= 8.2 Hz, 1H), 5.92–5.78 (m, 1H), 5.11 (dd, *J* = 17.1, 1.6 Hz, 1H), 5.03 (dd, *J* = 10.1, 1.5 Hz, 1H), 4.64 (t, *J* = 7.2 Hz, 1H), 3.66 (s, 3H), 2.76–2.69 (m, 2H), 2.52 (s, 3H); ^13^C NMR (100 MHz, CDCl_3_) *δ* 195.39, 170.86, 156.84, 149.94, 143.37, 135.10, 127.48, 119.46, 117.62, 54.80, 52.62, 32.76, 24.11.

#### 4.1.5. Methyl 2-[6-methyl-3-(prop-1-en-2-yl)picolinoyl]pent-4-enoate (**11**)

To a solution of methyl 2-(3-bromo-6-methylpicolinoyl)pent-4-enoate (1.04 g, 3.34 mmol) in 1,4-dioxane/water (32 ml, 3/1, *v/v)* were added sodium carbonate (1.08 g, 10.2 mmol, 3.00 eq.), Pd(PPh_3_)_4_ (0.38 g, 0.33 mmol, 0.10 eq.), and isopropenylboronic acid pinacol ester (0.75 ml, 4.01 mmol, 1.20 eq.), then the mixture was stirred at reflux for 4 h under N_2_ atmosphere. Then 10.0 ml of water was added to the reaction mixture. The mixture was then partitioned between CH_2_Cl_2_ (50.0 ml) and 2 M aqueous sodium carbonate. The aqueous layer was extracted two more times with CH_2_Cl_2_ (30.0 ml), and the combined organic portions were dried over magnesium sulfate, filtered and concentrated *in vacuo*. The resulted residue was purified by silica gel column chromatography (ethyl acetate/petroleum, 1/15) to obtain the title compound (0.84 g, 91.8%) as a yellow oil. HR-ESI-MS, Calcd 273.1365, found [M + H]^+^ = 274.1428; IR (neat, film) *v*_max_ 3079, 2980, 2950, 2848, 1744, 1710, 1640, 1588, 1435, 1294, 1251, 1193, 915, 839, 699 cm^−1^; ^1^H NMR (400 MHz, CDCl_3_) *δ* 7.45 (d, *J* = 7.9 Hz, 1H), 7.23 (d, *J* = 7.8 Hz, 1H), 5.85 (ddt, *J* = 17.0, 10.2, 6.8 Hz, 1H), 5.12 (d, *J* = 1.6 Hz, 1H), 5.00 (dd, *J* = 10.0, 1.7 Hz, 1H), 4.81 (d, *J* = 0.9 Hz, 1H), 4.74 (t, *J* = 7.2 Hz, 1H), 3.65 (s, 3H), 2.76–2.68 (m, 2H), 2.54 (s, 3H), 2.03 (d, *J* = 1.2 Hz, 3H); ^13^C NMR (100 MHz, CDCl_3_) *δ* 196.66, 170.96, 156.04, 148.76, 138.58, 135.00, 132.71, 125.94, 118.54, 116.82, 114.23, 54.04, 51.93, 36.94, 32.44, 23.53.

#### 4.1.6. Methyl 9-hydroxy-2,5-dimethyl-7*H*-cyclohepta[*b*]pyridine-8-carboxylate (**13**)

To a solution of methyl 2-[6-methyl-3-(prop-1-en-2-yl)picolinoyl]pent-4-enoate (1.00 g, 3.67 mmol) in CH_2_Cl_2_ (30.0 ml) was added Grubbs catalyst II (0.31 g, 0.37 mmol, 10% mol) and then the mixture was stirred at reflux for 12 h. Afterwards the reaction mixture was concentrated *in vacuo*. The resulting residue was purified by silica gel column chromatography (ethyl acetate/petroleum, 1/5) to obtain the title compound (0.47 g, 53.3%) as a yellow oil. HR-ESI-MS, Calcd 245.1052, found [M + H]^+^ = 246.1118; IR (neat, film) *v*_max_ 3020, 2922, 2851, 1646, 1611, 1587, 1441, 1232, 1166, 792, 667 cm^−1^; ^1^H NMR (400 MHz, CDCl_3_) *δ* 12.35 (s, 1H), 7.76 (d, *J *= 8.2 Hz, 1H), 7.26 (d, *J* = 8.2 Hz, 1H), 6.08 (t, *J* = 7.4 Hz, 1H), 3.85 (s, 3H), 2.69 (s, 3H), 2.52 (d, *J* = 7.0 Hz, 2H), 2.09 (s, 3H); ^13^C NMR (100 MHz, CDCl_3_) *δ* 171.92, 165.08, 157.15, 149.75, 135.61, 134.96, 134.75, 132.44, 130.65, 124.26, 106.05, 52.54, 31.38, 24.93, 21.88.

#### 4.1.7. Methyl 9-hydroxy-2,5-dimethyl-8,9-dihydro-7*H*-cyclohepta[*b*]pyridine-8-carboxylate (**15**)

To a solution of methyl 9-hydroxy-2,5-dimethyl-7*H*-cyclohepta[*b*]pyridine-8-carboxylate (100 mg, 0.40 mmol) in methanol (10.0 ml) was added NaBH_4_ (18.6 mg, 0.48 mmol, 1.20 eq.). The mixture was stirred for 30 min in an ice bath, then the reaction was quenched with water (5.00 ml) and evaporated to dryness. The resulting residue was dissolved in pyridine (10.0 ml) and methanesulfonyl chloride (5.50 mg, 0.48 mmol, 1.20 eq.) was added. The mixture was stirred for 3 h at 60°C. The reaction was quenched with water (5.00 ml), extracted with CH_2_Cl_2_ (10.0 ml × 3), and the combined extracts were dried over magnesium sulfate, filtered and concentrated *in vacuo*. The resulting residue was purified by silica gel column chromatography (ethyl acetate/petroleum, 1/5) to give the title compound (61.4 mg, 67.1%) as a yellow oil. HR-ESI-MS, Calcd 229.1103, found [M + H]^+^ = 230.1167; IR (neat, film) *v*_max_ 3020, 2949, 2850, 1711, 1636, 1586, 1434, 1194, 1092, 836, 760 cm^−1^; ^1^H NMR (400 MHz, CDCl_3_) *δ* 7.80 (d, *J* = 8.2 Hz, 1H), 7.74 (s, 1H), 7.15 (d, *J* = 8.2 Hz, 1H), 5.85 (t, *J* = 7.3 Hz, 1H), 3.82 (s, 3H), 2.67 (d, *J* = 7.2 Hz, 2H), 2.62 (s, 3H), 2.11 (s, 3H); ^13^C NMR (100 MHz, CDCl_3_) *δ* 167.15, 156.77, 152.60, 138.30, 135.85, 135.17, 134.96, 133.33, 126.87, 122.27, 52.60, 24.95, 24.77, 22.29.

#### 4.1.8. Methyl 2,5-dimethyl-6,7,8,9-tetrahydro-5*H*-cyclohepta[*b*]pyridine-8-carboxylate (**16**)

To a solution of methyl 9-hydroxy-2,5-dimethyl-8,9-dihydro-7*H*-cyclohepta[*b*]pyridine-8-carboxylate (50.0 mg, 0.22 mmol) in methanol (5.00 ml) was added Pd/C (40.0 mg, 0.5% Pd in C). The mixture was stirred for 5 h under H_2_ in room temperature. After filtration of Pd/C, the reaction mixture was concentrated *in vacuo* to give rupestine **G** and its epimers (46.4 mg, 91.4%) as a colourless oil. The mixture (46.4 mg) was firstly isolated on a preparative TLC to give two pairs of diastereoisomers **16a** (31.0 mg) and **16b** (10.6 mg). These two pairs of compounds were further separated by chiral separation with a Shimadzu LC-20A preparative-HPLC (CHIRALPAK ID-Lot No. ID00CE-QI011 used as chiral column, *n*-hexane/ethanol (98/2, *v/v*) used as mobile phase) to give the four optically pure isomers.

#### 4.1.9. Methyl (5*S*,8*S*)-2,5-dimethyl-6,7,8,9-tetrahydro-5*H*-cyclohepta[*b*]pyridine-8-carboxylate (**5A**, aka, rupestine **G**)

Colourless oil; [*α*]_D_^20^ = −41.0 (c = 0.10, MeOH); HR-ESI-MS, Calcd 233.1416, found [M + H]^+^ = 234.1490. IR (neat, film) *v*_max_ 2977, 2882, 1733, 1593, 1473, 1188, 1158, 803 cm^−1^. ^1^H NMR (600 MHz, CDCl_3_) *δ*6.92 (d, *J *= 7.7 Hz, 1H, 3-H), 7.30 (d, *J* = 7.7 Hz, 1H, 4-H), 3.02–2.95 (m, 1H, 5-H), 1.85–1.79 (m, 1H, 6-α-H), 1.78–1.74 (m, 1H, 6-β-H), 1.99–1.94 (m, 1H, 7-α-H), 2.15–2.08 (m, 1H, 7-β-H), 2.64 (t, *J* = 9.8 Hz, 1H, 8-H), 3.28 (dd, *J *= 14.6, 3.3 Hz, 1H, 9-α-H), 3.37 (dd, *J* = 14.6, 9.9 Hz, 1H, 9-β-H), 1.31 (d, *J *= 7.3 Hz, 3H, 13-H), 3.64 (s, 3H, 14-H), 2.48 (s, 3H, 15-H); ^13^C NMR (150 MHz, CDCl_3_) *δ* 157.30 (C-2), 121.23 (C-3), 136.10 (C-4), 37.54 (C-5), 32.16 (C-6), 29.04 (C-7), 41.94 (C-8), 40.43 (C-9), 154.60 (C-10), 137.74 (C-11), 175.60 (C-12), 18.76 (C-13), 51.51 (C-14), 23.74 (C-15).

#### 4.1.10. Methyl (5*R*,8*R*)-2,5-dimethyl-6,7,8,9-tetrahydro-5*H*-cyclohepta[*b*]pyridine-8-carboxylate (**5B**)

Colourless oil; [*α*]_D_^20^ = +39.0 (c = 0.10, MeOH); HR-ESI-MS, Calcd 233.1416, found [M + H]^+^ = 234.1489. IR (neat, film) *v*_max_ 2986, 2883, 1734, 1608, 1458, 1188, 1158, 800 cm^−1. 1^H NMR (400 MHz, CDCl_3_) *δ* 6.93 (d, *J* = 7.7 Hz, 1H, 3-H), 7.31 (d, *J* = 7.7 Hz, 1H, 4-H), 3.04–2.95 (m, 1H, 5-H), 1.86–1.74 (m, 2H, 6-H), 2.01–1.93 (m, 1H, 7-α-H), 2.17–2.07 (m, 1H, 7-β-H), 2.70–2.61 (m, 1H, 8-H), 3.31 (d, *J* = 14.6, 2.7 Hz, 1H, 9-*α*-H), 3.36 (dd, *J* = 14.6, 9.7 Hz, 1H, 9-*β*-H), 1.32 (d, *J* = 7.3 Hz, 3H, 13-H), 3.64 (s, 3H, 14-H), 2.49 (s, 3H, 15-H); ^13^C NMR (100 MHz, CDCl_3_) *δ* 157.19 (C-2), 121.16 (C-3), 136.05 (C-4), 37.41 (C-5), 32.05 (C-6), 28.96 (C-7), 41.80 (C-8), 40.26 (C-9), 154.46 (C-10), 137.68 (C-11), 175.49 (C-12), 51.43 (C-13), 18.66 (C-14), 23.60 (C-15).

#### 4.1.11. Methyl (5*R*,8*S*)-2,5-dimethyl-6,7,8,9-tetrahydro-5*H*-cyclohepta[*b*]pyridine-8-carboxylate (**5C**)

Colourless oil; [*α*]_D_^20 ^= +17.0 (c = 0.10, MeOH); HR-ESI-MS, Calcd 233.1416, found [M + H]^+^ = 234.1490. IR (neat, film) *v*_max_ 2945, 2883, 1743, 1608, 1458, 1188, 1158, 821 cm^−1^. ^1^H NMR (600 MHz, CDCl_3_) *δ*6.99 (d, *J* = 7.9 Hz, 1H, 3-H), 7.38 (d, *J* = 7.9 Hz, 1H, 4-H), 3.05–2.94 (m, 1H, 5-H), 1.91–1.86 (m, 1H, 6-α-H), 1.29–1.24 (m, 1H, 6-β-H), 2.01–1.94 (m, 1H, 7-α-H), 2.17–2.12 (m, 1H, 7-β-H), 2.49–2.44 (m, 1H, 8-H), 3.34–3.25 (m, 2H, 9-H), 1.34 (d, *J* = 7.1 Hz, 3H, 13-H), 3.69 (s, 3H, 14-H), 2.50 (s, 3H, 15-H); ^13^C NMR (150 MHz, CDCl_3_) *δ*158.89 (C-2), 121.43 (C-3), 132.71 (C-4), 35.08 (C-5), 33.93 (C-6), 29.85 (C-7), 42.23 (C-8), 40.55 (C-9), 154.59 (C-10), 138.00 (C-11), 176.21 (C-12), 51.90 (C-13), 20.48 (C-14), 23.91 (C-15).

#### 4.1.12. Methyl (5*S*,8*R*)-2,5-dimethyl-6,7,8,9-tetrahydro-5*H*-cyclohepta[*b*]pyridine-8-carboxylate (**5D**)

Colourless oil; [*α*]_D_^20 ^= −18.0 (c = 0.10, MeOH); HR-ESI-MS, Calcd 233.1416, found [M + H]^+^ = 234.1490. IR (neat, film) *v*_max_ 2944, 2883, 1734, 1593, 1473, 1188, 1158, 803 cm^−1^. ^1^H NMR (600 MHz, CDCl_3_) *δ* 6.99 (d, *J* = 7.9 Hz, 1H, 3-H), 7.38 (d, *J* = 7.9 Hz, 1H, 4-H), 3.05–2.94 (m, 1H, 5-H), 1.92–1.85 (m, 1H, 6-α-H), 1.27–1.24 (m, 1H, 6-β-H), 2.02–1.93 (m, 1H, 7-α-H), 2.17–2.11 (m, 1H, 7-β-H), 2.49–2.44 (m, 1H, 8-H), 3.34–3.23 (m, 2H, 9-H), 1.34 (d, *J* = 7.0 Hz, 3H, 13-H), 3.69 (s, 3H, 14-H), 2.50 (s, 3H, 15-H); ^13^C NMR (150 MHz, CDCl_3_) *δ* 159.11 (C-2), 121.62 (C-3), 132.86 (C-4), 35.31 (C-5), 35.21 (C-6), 34.15 (C-7), 42.45 (C-8), 40.67 (C-9), 154.84 (C-10), 138.19 (C-11), 176.44 (C-12), 52.11 (C-13), 20.70 (C-14), 24.16 (C-15).

## Supplementary Material

Structures of all natural rupestines, NMR spectrum of compounds 8~15, 5A~5D, experimental ECD and calculated ECD of compounds 5A~5D, optical rotation spectrum of compounds 5A~5D

## Data accessibility

Structures of all natural rupestines, NMR spectrum of compounds **8–15**, **5A–5D**, experimental and calculated ECD of compounds **5A–5D**, optical rotation spectrum of compounds **5A–5D** associated with this paper can be found in the electronic supplementary material.
