# Supplementary material for: Total synthesis of rupestine G and its epimers
Source: R Soc Open Sci. 2018 Mar 28;5(3):172037. doi: 10.1098/rsos.172037 (PMC5882726; doi:10.1098/rsos.172037)
Supplement: Structures of all natural rupestines, NMR spectrum of compounds 8~15, 5A~5D, experimental ECD and calculated ECD of compounds 5A~5D, optical rotation spectrum of compounds 5A~5D [file rsos172037supp1.docx]

Electronic Supplement Material

Total Synthesis of Rupestine G and its Epimers

Abdullah Yusuf ^1,2,3^, Jiangyu Zhao ^1^, Bianlin Wang ^1^, Paruke Aibibula^1,2^,

Haji Akber Aisa ^1,^ Guozheng Huang ^1,^*

^1^ Key Laboratory of Plant Resources and Chemistry in Arid Regions, and State Key Laboratory Basis of Xinjiang Indigenous Medicinal Plants Resource Utilization, Xinjiang Technical Institute of Physics and Chemistry, Chinese Academy of Sciences, South Beijing Rd 40-1, Urumqi, 830011, P. R. China;

^2^ University of Chinese Academy of Sciences, Yuquan Rd 19 A, Beijing, 100049, P. R. China;

^3^ College of Chemistry and Environmental Science, Kashgar University, Xueyuan Rd 29, Kashgar, 844000, P. R. China;

* corresponding author. E-mail: guozheng.huang@yahoo.com, [g.huang@ms.xjb.ac.cn](mailto:g.huang@ms.xjb.ac.cn) ; Tel: +86-991-3836733 (G.Huang)

**Figure S0：**Structures of rupestines.

Rupestine **B** and **C,** rupestine **D** and **P**, rupestine **H** and **I**, rupestine **K** and **O**,as well as rupestine **L** and **M**, are natural isomeric compounds. Rupestine **E** was once erroneously assigned as its (*5R*,*8R*) - isomer, i.e. rupestine, which has not yet isolated from the natural plant. (c.f. He F. **2012**, Studies on the chemical constituents and HPLC fingerprint of *Artemisia rupestris L*. in Xinjiang. [D]. Xinjiang Technical Institute of Physics and Chemistry, Chinese Academy of Sciences. Urumqi, China)

**Spectral Data of the intermediates 8~15**

**Figure S1：**^1^H NMR (400 MHz, CDCl_3_) spectrum of 3-bromo-6-methylpicolinonitrile (**8**)

**Figure S2：**HR-ESI-MS spectrum of 3-bromo-6-methylpicolinonitrile (**8**)

**Figure S3：**^1^H NMR (400 MHz, CDCl_3_) spectrum of methyl 3-(3-bromo-6-methylpyridin-2-yl)-3-oxopropanoate (**9**)

**Figure S4：**^13^C NMR (100 MHz, CDCl_3_) spectrum of methyl 3-(3-bromo-6-methylpyridin-2-yl)-3-oxopropanoate (**9**)

**Figure S5：**HR-ESI-MS spectrum of methyl 3-(3-bromo-6-methylpyridin-2-yl)-3-oxopropanoate (**9**)

**Figure S6：**^1^H NMR (400 MHz, CDCl_3_) spectrum of methyl 2-(3-bromo-6-methylpicolinoyl) pent-4-enoate (**10**)

**Figure S7：**^13^C NMR (100 MHz, CDCl_3_) spectrum of methyl 2-(3-bromo-6-methylpicolinoyl) pent-4-enoate (**10**)

**Figure S8：**HR-ESI-MS spectrum of methyl 2-(3-bromo-6-methylpicolinoyl) pent-4-enoate (**10**)

**Figure S9：**^1^H NMR (400 MHz, CDCl_3_) spectrum of methyl 2-[6-methyl-3-(prop-1-en-2-yl)picolinoyl]pent-4-enoate (**11**)

**Figure S10：**^13^C NMR (100 MHz, CDCl_3_) spectrum of methyl 2-[6-methyl-3-(prop-1-en-2-yl)picolinoyl]pent-4-enoate (**11**)

**Figure S11：**HR-ESI-MS spectrum of methyl 2-[6-methyl-3-(prop-1-en-2-yl)picolinoyl]pent-4-enoate (**11**)

**Figure S12：**^1^H NMR (400 MHz, CDCl_3_) spectrum of methyl 9-hydroxy-2,5-dimethyl-7*H*-cyclohepta[*b*]pyridine- 8-carboxylate (**13**)

**Figure S13：**^13^C NMR (100 MHz, CDCl_3_) spectrum of methyl 9-hydroxy-2,5-dimethyl-7*H*-cyclohepta[*b*]pyridine- 8-carboxylate (**13**)

**Figure S14：**HR-ESI-MS spectrum of methyl 9-hydroxy-2,5-dimethyl-7*H*-cyclohepta[*b*]pyridine-8-carboxylate (**13**)

**Figure S15：**^1^H NMR (400 MHz, CDCl_3_) spectrum of methyl 2,5-dimethyl-7*H*-cyclohepta[*b*]pyridine-8-carboxylate (**15**)

**Figure S16：**^13^C NMR (100 MHz, CDCl_3_) spectrum of methyl 2,5-dimethyl-7*H*-cyclohepta[*b*]pyridine-8-carboxylate (**15**)

**Figure S17：**HR-ESI-MS spectrum of methyl 2,5-dimethyl-7*H*-cyclohepta[*b*]pyridine-8-carboxylate (**15**)

**NMR, HR-ESI-MS, ECD and Optical rotation spectrum of rupestine G and its epimers**

**Figure S18：**^1^H NMR (600 MHz, CDCl_3_) spectrum of (5*S*,8*S*)-methyl-2,5-dimethyl-6,7,8,9-tetrahydro-5*H*-cyclohepta[*b*] pyridine-8-carboxylate (**5A**)

**Figure S19：**^13^C NMR (150 MHz, CDCl_3_) spectrum of (5*S*,8*S*)-methyl-2,5-dimethyl-6,7,8,9-tetrahydro-5*H*-cyclohepta[*b*] pyridine-8-carboxylate (**5A**)

**Figure S20：**HR-ESI-MS spectrum of (5*S*,8*S*)-methyl-2,5-dimethyl-6,7,8,9-tetrahydro-5*H*-cyclohepta[*b*]pyridine- 8-carboxylate (**5A**)

**Figure S21：**^1^H NMR (400 MHz, CDCl_3_) spectrum of (5*R*,8*R*)-methyl-2,5-dimethyl-6,7,8,9-tetrahydro-5*H*-cyclohepta[*b*] pyridine-8-carboxylate (**5B**)

**Figure S22：**^13^C NMR (100 MHz, CDCl_3_) spectrum of (5*R*,8*R*)-methyl-2,5-dimethyl-6,7,8,9-tetrahydro-5*H*-cyclohepta[*b*] pyridine-8-carboxylate (**5B**)

**Figure S23：**HR-ESI-MS spectrum of (5*R*,8*R*)-methyl-2,5-dimethyl-6,7,8,9-tetrahydro-5*H*-cyclohepta[*b*]pyridine- 8-carboxylate (**5B**)

**Figure S24：**^1^H NMR (600 MHz, CDCl_3_) spectrum of (5*R*,8*S*)-methyl-2,5-dimethyl-6,7,8,9-tetrahydro-5*H*-cyclohepta[*b*] pyridine-8-carboxylate (**5C**)

**Figure S25：**^13^C NMR (150 MHz, CDCl_3_) spectrum of (5*R,*8*S*)-methyl-2,5-dimethyl-6,7,8,9-tetrahydro-5*H*-cyclohepta[*b*] pyridine-8-carboxylate (**5C**)

**Figure S26：**HR-ESI-MS spectrum of (5*R,*8*S*)-methyl-2,5-dimethyl-6,7,8,9-tetrahydro-5*H*-cyclohepta[*b*]pyridine- 8-carboxylate (**5C**)

**Figure S27：**^1^H NMR (600 MHz, CDCl_3_) spectrum of (5*S,*8*R*)-methyl-2,5-dimethyl-6,7,8,9-tetrahydro-5*H*-cyclohepta[*b*] pyridine-8-carboxylate (**5D**)

**Figure S28：**^13^C NMR (150 MHz, CDCl_3_) spectrum of (5*S,*8*R*)-methyl-2,5-dimethyl-6,7,8,9-tetrahydro-5*H*-cyclohepta[*b*] pyridine-8-carboxylate (**5D**)

**Figure S29：**HR-ESI-MS spectrum of (5*S,*8*R*)-methyl-2,5-dimethyl-6,7,8,9-tetrahydro-5*H*-cyclohepta[*b*]pyridine- 8-carboxylate (**5D**)

**Figure S30：**The experimental ECD and calculated ECD of (5*S,*8*S*)-methyl-2,5-dimethyl-6,7,8,9-tetrahydro-5*H*-cyclohepta [*b*]pyridine-8-carboxylate(**5A**) and (5*R,*8*R*)-methyl-2,5-dimethyl-6,7,8,9-tetrahydro-5*H*-cyclohepta[*b*]pyridine-8- carboxylate(**5B**)

**Figure S31：**The experimental ECD and calculated ECD of (5*R,*8*S*)-methyl-2,5-dimethyl-6,7,8,9-tetrahydro-5*H*-cyclohepta [*b*]pyridine-8-carboxylate(**5C**) and (5*S,*8*R*)-methyl-2,5-dimethyl-6,7,8,9-tetrahydro-5*H*-cyclohepta[*b*]pyridine-8- carboxylate(**5D**)

Circular dichroism (CD) spectroscopy and time dependent density functional theory (TDDFT) calculations at B3LYP/TZVPP level. ECD spectra were measured in EtOH on a JASCO J – 810 spectropolarimeter (Jasco, Tokyo, Japan). The calculations of ECD spectra were performed by TmoleX 3.4 software (COSMO logic GmbH & Co. KG, Germany).


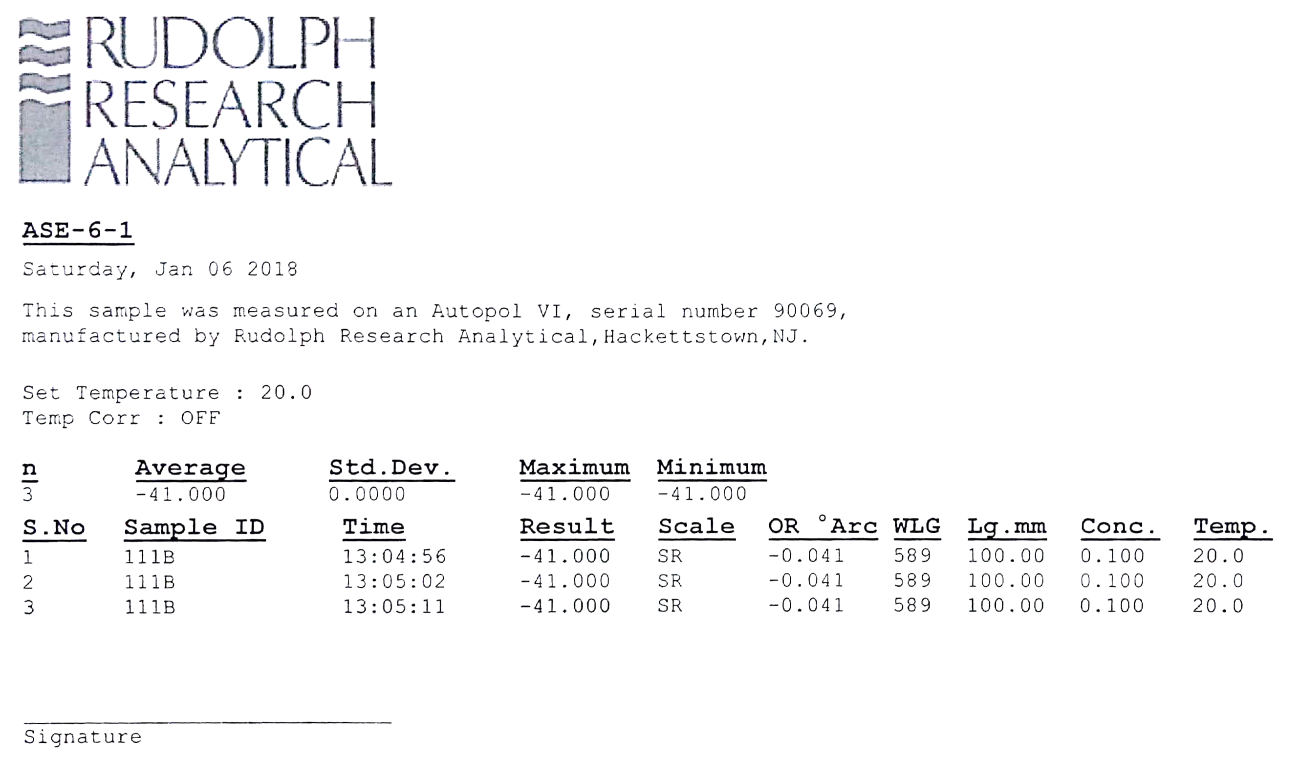


**Figure S32：**Optical rotation spectrum of (5*S,*8*S*)-methyl-2,5-dimethyl-6,7,8,9-tetrahydro-5*H*-cyclohepta[*b*]pyridine- 8-carboxylate (**5A**)


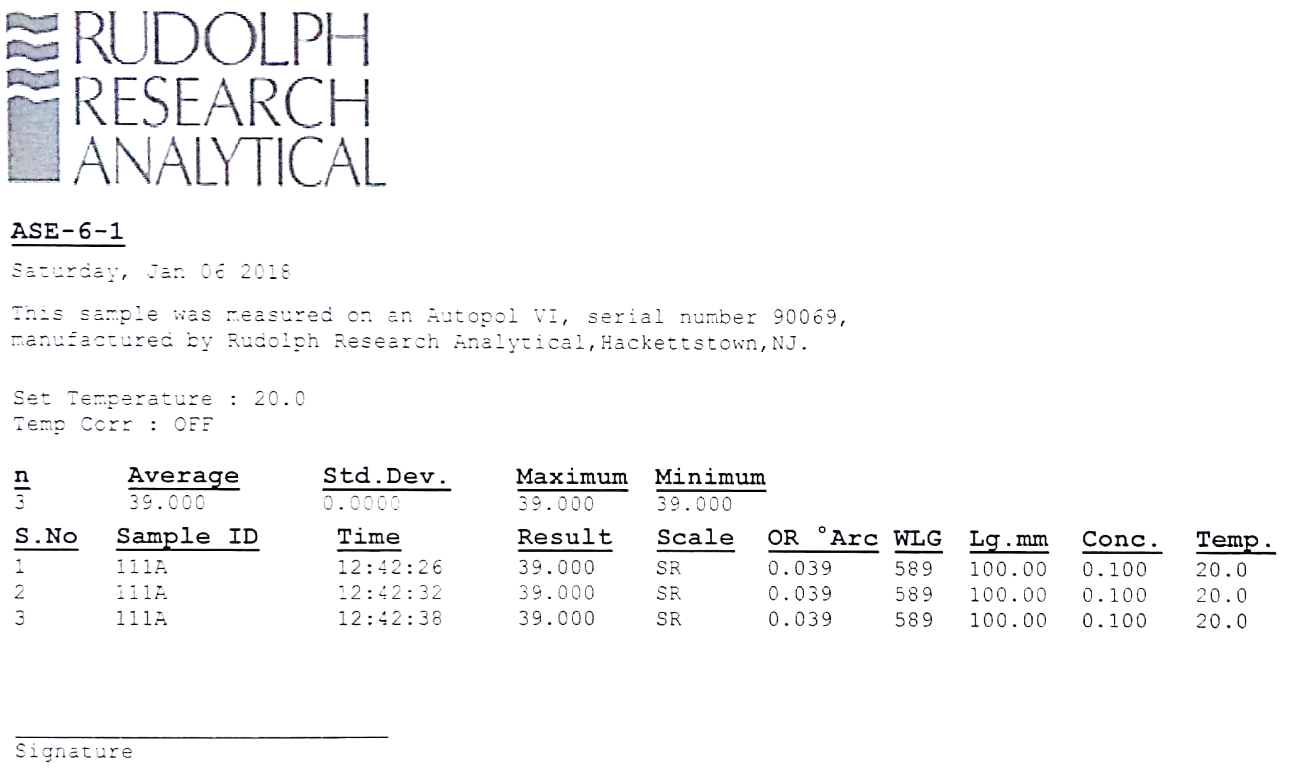


**Figure S33：**Optical rotation spectrum of (5*R,*8*R*)-methyl-2,5-dimethyl-6,7,8,9-tetrahydro-5*H*-cyclohepta[*b*]pyridine- 8-carboxylate (**5B**)


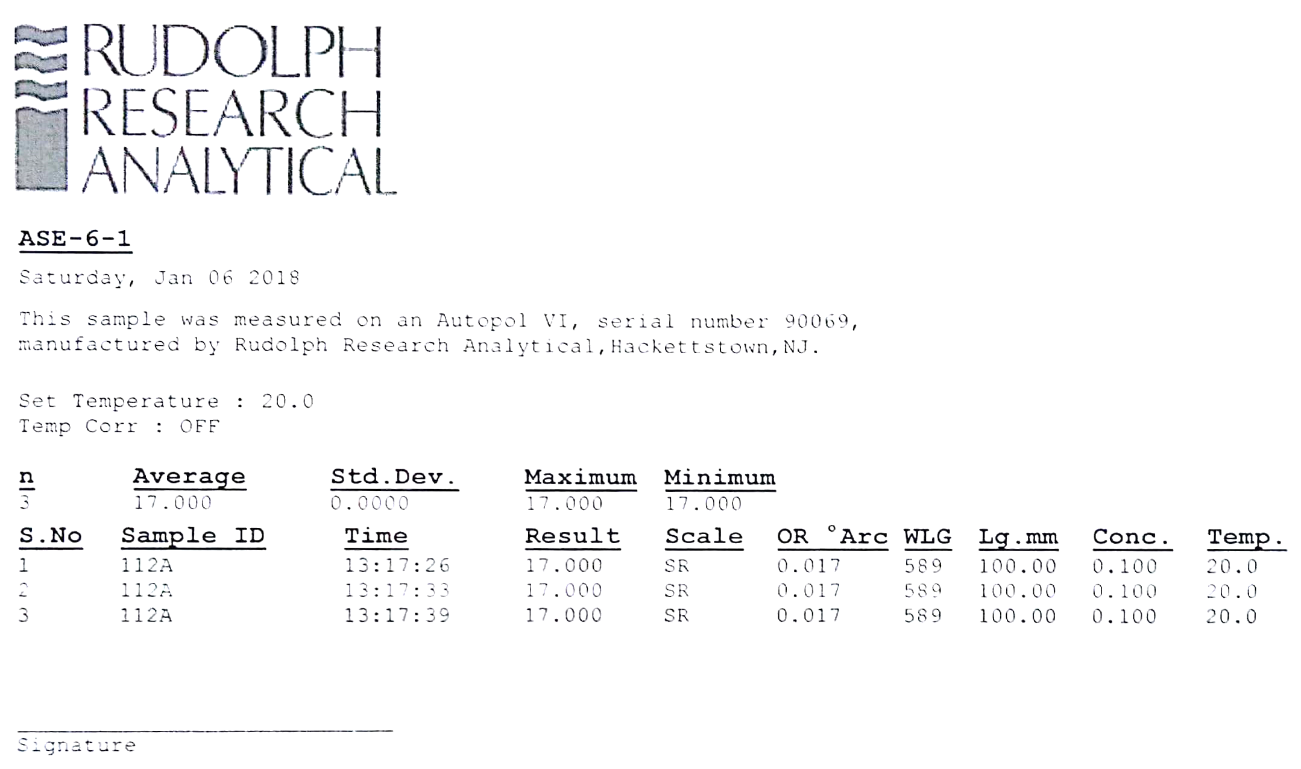


**Figure S34：**Optical rotation spectrum of (5*R,*8*S*)-methyl-2,5-dimethyl-6,7,8,9-tetrahydro-5*H*-cyclohepta[*b*]pyridine- 8-carboxylate (**5C**)


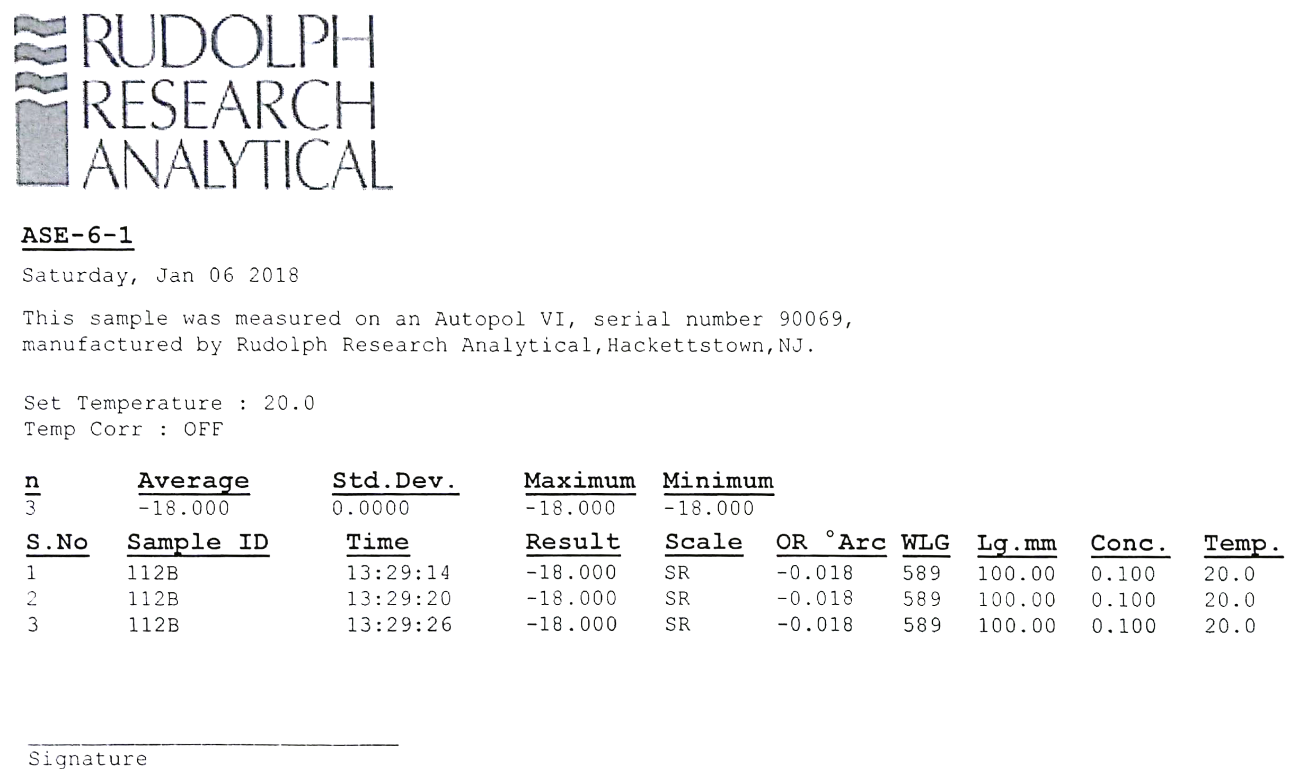


**Figure S35：**Optical rotation spectrum of (5*S,*8*R*)-methyl-2,5-dimethyl-6,7,8,9-tetrahydro-5*H*-cyclohepta[*b*]pyridine- 8-carboxylate (**5D**)
